# Einleitung zum Forum: Die Corona-Pandemie – Konflikt und Frieden in einer veränderten Welt

**DOI:** 10.1007/s42597-020-00053-x

**Published:** 2020-11-19

**Authors:** Hartwig Hummel, Solveig Richter, Alexander Spencer

**Affiliations:** 1 Institut für Sozialwissenschaften, Heinrich-Heine-Universität Düsseldorf, Universitätsstr. 1, 40225 Düsseldorf, Deutschland; 2 Institut für Politikwissenschaft, Universität Leipzig, Beethovenstraße 15, 04107 Leipzig, Deutschland; 3 Institut für Gesellschaftswissenschaften, Otto-von-Guericke-Universität Magdeburg, Universitätsplatz 2, 39106 Magdeburg, Deutschland

Die Corona-Pandemie hat weitreichende gesellschaftliche, politische, ökonomische und kulturelle Auswirkungen auf lokaler, nationaler und globaler Ebene. Auch die Friedens- und Konfliktforschung ist aufgerufen, sich zu dieser historischen und die gesamte Menschheit betreffenden Zäsur zu äußern. Als Herausgeber*innen der ZeFKo haben wir uns daher entschlossen, ein Forum zum Thema „Die Corona-Pandemie: Konflikt und Frieden in einer veränderten Welt“ für dieses Heft zu organisieren. Die Rubrik „Forum“ dient unter anderem dazu, ein aktuelles Thema für die Friedens- und Konfliktforschung aus unterschiedlichen Blickwinkeln zu betrachten, intensiv zu diskutieren und dadurch weitere Diskussionen anzustoßen.

Anfang April starteten wir über die AFK und weitere einschlägige Netzwerke einen entsprechenden Call für Forumsbeiträge, der auf große Resonanz stieß. In einem zweistufigen Verfahren baten wir zunächst um die Zusendung kurzer Abstracts. Aus den 27 Einsendungen wählten wir dann diejenigen Vorschläge aus, die an wissenschaftliche Debatten in der Friedens- und Konfliktforschung anschlossen, also über persönliche oder politische Statements hinausgingen, und die bereits soweit ausgearbeitet waren, dass daraus in der kurzen verfügbaren Zeit substanzielle Forumsbeiträge zu erwarten waren. Alle Beiträge durchliefen einen internen Begutachtungsprozess. Auch wenn unter den gegebenen Umständen nur ein Teil der Vorschläge realisiert werden konnte, möchten wir uns ganz herzlich bei allen Kolleg*innen, die uns ihre Vorschläge geschickt haben, bedanken. Im Ergebnis können wir in diesem Forum 10 Beiträge veröffentlichen, die die Breite, Kreativität und Relevanz der Friedens- und Konfliktforschung eindrucksvoll abbilden. Es handelt sich um Beiträge sowohl aus Forschungsinstituten, als auch aus Universitäten, von Nachwuchswissenschaftler*innen wie von etablierten Professor*innen. Die Verankerung in der deutschsprachigen Community, gleichzeitig aber auch die internationale Anschlussfähigkeit zeigt sich darin, dass die Beiträge jeweils zur Hälfte in deutscher und in englischer Sprache verfasst sind.

Die Forumsbeiträge behandeln vier Themenbereiche:

## Corona und Sicherheit

*Elena Sondermann und Cornelia Ulbert* (Institut für Entwicklung und Frieden, Universität Duisburg-Essen) argumentieren, dass die Metapher des „Krieges gegen das Coronavirus“ zwar die Dringlichkeit von Gegenmaßnahmen unterstreicht, jedoch komplexe Linien von Kausalität und Verantwortung verkürzt und nationale Antworten privilegiert. Sie stellen einer solchen „Logik des Ausnahmezustands“ eine alternative Erzählung gegenüber, die auf der „Logik der Solidarität“ basiert, und die Option einer nachhaltigeren und gerechteren Art der Bewältigung von Infektionskrankheiten eröffnet.

Der UN-Sicherheit verabschiedete im Jahr 2000 die Resolution 1325 zum Thema „Frauen, Frieden und Sicherheit“ als Konkretisierung des Konzepts der menschlichen Sicherheit. Vor dem Hintergrund dieser globalen Agenda diskutiert *Manuela Scheuermann *(Institut für Politikwissenschaft und Soziologie, Julius-Maximilians-Universität Würzburg) die beunruhigenden „gendered effects“ der Pandemie anhand empirischer Daten, die vor allem UN Women in Zusammenarbeit mit der Weltgesundheitsorganisation seit Ausbruch der Pandemie gesammelt hat.

*Una Jakob* (HSFK Frankfurt a.M.) nimmt Anschuldigungen, die Pandemie sei durch die absichtliche oder fahrlässige Freisetzung des neuartigen Corona-Virus herbeigeführt worden, zum Anlass, auf die Mängel bei der Verifikation des Verbotsregimes für biologische Waffen hinzuweisen. Sie stellt Möglichkeiten vor, wie das Biowaffenverbot besser umgesetzt werden könnte und wie dadurch gleichzeitig auch negative politische Dynamiken bei ungewöhnlichen Krankheitsausbrüchen einzuhegen wären.

## Die Beschränkung politischer Partizipation in Zeiten von Corona

*Tareq Sydiq* (Zentrum für Konfliktforschung, Philipps-Universität Marburg) erinnert an die großen oppositionelle Protestbewegungen weltweit, die vor Ausbruch der Pandemie großes öffentliches Interesse fanden. Der Beginn der Pandemie schien dieser politischen Dynamik schlagartig ein Ende zu setzen, sei es, dass sich die öffentliche Aufmerksamkeit nun auf die Pandemie konzentrierte, sei es wegen der Beschränkungen zur Pandemie-Bekämpfung. Der Autor weist jedoch auch auf neue Ausdrucks- und Organisationsformen des Protestes hin, deren Mobilisierungspotenzial sich erst mittelfristig zeigen werde.

*Felix S. Bethke und Jonas Wolff* (HSFK, Frankfurt a.M.) beschreiben die weltweiten Einschränkungen der Versammlungsfreiheit und weiterer politischer Partizipationsrechte in Reaktionen auf die COVID-19-Pandemie. Sie befürchten, dass diese Maßnahmen den bereits zuvor zu beobachtenden globalen Trend zur „Schrumpfung“ der zivilgesellschaftlichen Räume verstärken, und konkretisieren diese Befürchtungen anhand von Erfahrungen aus einzelnen Ländern.

Im Streit um die Corona-Strategie brach die Regierungskoalition im Kosovo auseinander. *Werner Distler* (Zentrum für Konfliktforschung, Philipps-Universität Marburg) zeigt, dass der Diskurs über den „Ausnahmezustand“ und die Pandemie nicht zum Zusammenrücken der politischen Akteure, sondern zu intensivierten Machtkämpfen geführt hat. Er konstatiert im Zusammenspiel von internen und externen Akteuren eine Schwächung demokratischer Institutionen und eine Stärkung der etablierten Eliten.

## Peacebuilding unter den Bedingungen der Pandemie

In Kolumbien droht der Ausbruch des Coronavirus die Umsetzung friedensbezogener Projekte zu verlangsamen, während gleichzeitig die Gewalt insbesondere über die Kontrolle und den Besitz von Land zunimmt. Vor diesem Hintergrund sucht das Team von *Luca Eufemia, Camilo Lozano, Tatiana Rodriguez, Martha Del Rio, Héctor Morales, Michelle Bonatti, Stefan Sieber und Katharina Löhr* (Leibniz-Zentrum für Agrarlandschaftsforschung) nach risikoadaptierten Strategien für entsprechende nationale und internationale Kooperations- und Entwicklungsprojekte im Rahmen der Friedenskonsolidierung. Sie weisen dabei besonders auf die Bedeutung lokaler Akteure hin, auf die öffentlich-private und ökologische Projekte aufbauen sollten.

*André Bank und Sabine Kurtenbach* (GIGA Hamburg) untersuchen an den Fällen Kolumbien und Syrien, wie die Corona-Krise die Dynamiken von Gewalt und Frieden nach bzw. am Ende von Bürgerkriegen beeinflusst. Sie bedauern, dass der Aufruf von UN-Generalsekretär António Guterres, angesichts der Bedrohung durch die Covid-19-Pandemie die Waffen ruhen zu lassen, wirkungslos geblieben ist. Die Pandemie-Krise habe nicht zum erhofften Einhalten und Umdenken der Konfliktparteien geführt, sondern bereits vor Ausbruch der Pandemie bestehende Konfliktdynamiken verstärkt und beschleunigt.

## Gesundheit und Menschenrechte

*Anne Jenichen* (Aston University, Birmingham) *und Andrea Schapper* (Stirling University, Stirling) diskutieren die völkerrechtliche Pflicht zur internationalen Zusammenarbeit bei der Verwirklichung sozialer Rechte wie des Rechts auf Gesundheit. Sie erörtern am Beispiel der Ebola-Ausbrüche in Afrika südlich der Sahara und der Impfallianz Gavi die Potenziale von Mehrebenen-Partnerschaften, in denen internationale Organisationen, nationale Regierungen und lokale nichtstaatliche Akteure ihre Ressourcen bündeln. Damit wollen sie leistungsfähigere Staaten an ihre Verpflichtung zur Zusammenarbeit erinnern, damit während der aktuellen Pandemie das Menschenrecht auf Gesundheit auch für schutzbedürftige Bevölkerungsgruppen in schwachen Staaten gewährleistet wird.

Im abschließenden Beitrag betonen *Christian Scheper und Carolina A. Vestena* (Institut für Entwicklung und Frieden, Universität Duisburg-Essen), dass sich in der Corona-Pandemie die bisherigen lückenhaften Ansätze, grundlegende Arbeitsstandards und Menschenrechte in globalen Lieferketten zu sichern und bestehende Konflikte zu befrieden, als unzureichend erwiesen haben. Der Beitrag verdeutlicht diese Problematik am Beispiel von Beschäftigten in globalen Lieferketten in Fabriken in Brasilien und Indien. Scheper und Vestena plädieren vor diesem Hintergrund für weitere Forschungen zu globalen Lieferketten im Rahmen der Friedens- und Konfliktforschung.

Wie im einleitenden Editorial zu diesem Heft bereits angekündigt, setzt Solveig Richter den in diesem Forum angestoßenen Diskussionsprozess im Dezember 2020 mit einem Autor*innen-Workshop an ihrer neuen Wirkungsstätte an der Universität Leipzig fort. Dies ist gleichzeitig der Einstand für das neue ZeFKo-Redaktionsteam in Leipzig, das das Team von Alexander Spencer in Magdeburg ablöst. Wir möchten uns auch bei dieser Gelegenheit bei beiden Redaktionsteams ganz herzlich für ihre engagierte Arbeit zur Verwirklichung dieses Corona-Forums und des anschließenden Workshops bedanken.

